# PD235 Impact Of A Consumer Panel To Inform Health Technology Assessments And Policy Development: The Singapore Experience

**DOI:** 10.1017/S0266462324004537

**Published:** 2025-01-07

**Authors:** Sok Huang Teo, Fiona Pearce, Ping-Tee Tan

## Abstract

**Introduction:**

Patient involvement in health technology assessment (HTA) at the organizational level is vital to drive process development and capacity-building, and to ensure patients have the same opportunities as other stakeholders to effect policy changes. This presentation discusses the role and impact of the Agency for Care Effectiveness (ACE) Consumer Panel in the co-development of patient involvement and education initiatives in Singapore.

**Methods:**

The composition and terms of reference of patient and citizen groups in overseas HTA or government agencies were reviewed to identify key aspects that could be generalized to Singapore’s context. Using selection criteria, 20 individuals from local patient or voluntary groups representing a broad range of health conditions were shortlisted; 15 were formally appointed to the Panel in April 2022. Their contributions and impact on ACE’s work and healthcare decision-making were documented throughout their two-year term. A qualitative survey was also conducted to seek members’ feedback on their participation in the Panel and identify areas to improve collaboration.

**Results:**

Since their appointment, ACE Consumer Panel members have played a key role in providing the collective voice of local patient organizations to guide ACE’s work and have co-developed processes that ensure meaningful patient involvement in policy development and HTA. They have also informed ACE’s workplan by providing advice on priority-setting, communication strategies, and patients’ information needs. During their term, processes to include patient input in HTAs were formalized leading to the co-development of a process guide and supporting fact sheets, a patient glossary, and two patient training modules, which will be continually updated based on patient feedback.

**Conclusions:**

The ACE Consumer Panel is the first long-standing engagement of healthcare consumers by Singapore’s Ministry of Health. The Panel’s contributions and impact on ACE’s work serve as an example for other decision-makers on how to meaningfully involve patients at the organizational level, to understand their priorities and preferences, and to ensure healthcare policies remain relevant for the people affected by them.

